# Severe Folate Deficiency Mimicking Myelodysplastic Syndrome/Acute Myeloid Leukemia: A Case Report

**DOI:** 10.1155/crh/8212282

**Published:** 2026-07-24

**Authors:** Scott Potter, Margaret Williams, Timothy M. Hanley

**Affiliations:** ^1^ Department of Pathology, Spencer Fox Eccles School of Medicine, University of Utah, Salt Lake City, Utah, USA, utah.edu; ^2^ ARUP Laboratories, Salt Lake City, Utah, USA, aruplab.com

**Keywords:** acute myeloid leukemia (AML), case report, folate deficiency, megaloblastic anemia, myelodysplastic syndrome/neoplasm (MDS)

## Abstract

Folate deficiency is common and often asymptomatic, but severe cases can cause megaloblastic anemia. Rarely, it presents with pancytopenia and bone marrow changes resembling myelodysplastic syndrome/neoplasm (MDS) and/or acute myeloid leukemia (AML), complicating diagnosis and management. We present a case of a patient with severe folate deficiency, in the setting of normal vitamin B12 levels, who presented with hematologic and bone marrow findings initially suggestive of MDS/AML that resolved with folate supplementation. This case highlights that the clinical and morphologic findings associated with severe folate deficiency can closely resemble those of high‐risk MDS or MDS/AML. Careful diagnostic evaluation, including nutritional assessment, is essential to avoid the misclassification of reversible nutritional deficiencies as myeloid neoplasms requiring cytotoxic therapy.

## 1. Introduction

Folate (vitamin B9) plays an indispensable role in hematopoiesis through its function in one‐carbon metabolism, which is essential for the synthesis of purines and thymidylate required for DNA replication and repair [1, 2]. Impaired purine and thymidine synthesis leads to defective DNA replication and decreased cellular division. This is of particular importance in rapidly proliferating tissues such as the bone marrow. While uncomplicated folate deficiency often presents with isolated anemia, severe folate deficiency with concurrent vitamin B12 deficiency may lead to profound pancytopenia and bone marrow morphologic changes that can closely mimic primary hematologic malignancies, such as myelodysplastic neoplasm (MDS) or even acute myeloid leukemia (AML), on initial clinical evaluation [3–5].

There are several causes of folate deficiency, including inadequate dietary intake, malabsorption syndromes, increased metabolic demand (e.g., pregnancy), chronic alcoholism, and medication effects (e.g., antimetabolites such as methotrexate). Unlike vitamin B12 stores, which can last for years, folate stores are relatively small and can be depleted within months, leading to overt deficiency if not corrected. Typically, uncomplicated folate deficiency manifests with symptoms of anemia, including fatigue, pallor, and dyspnea, accompanied by laboratory evidence of macrocytosis (elevated mean corpuscular volume [MCV]) and hypersegmented neutrophils [6, 7]. With progressive deficiency, bone marrow findings may include hypercellularity with erythroid hyperplasia, nuclear‐cytoplasmic asynchrony, and an increased proportion of large, immature precursors (megaloblastic change) [8]. In rare, severe cases, the resulting cytopenias and dysplastic features may be indistinguishable from those seen in primary hematologic neoplasms such as MDS or AML [3–5]. This potential for misclassification highlights the critical need for a thorough differential diagnosis that includes reversible nutritional deficiencies prior to diagnosing a hematologic malignancy.

MDS are clonal hematopoietic disorders characterized by ineffective hematopoiesis, peripheral cytopenias, morphologic dysplasia, and clonal cytogenetic or molecular abnormalities [9]. Additionally, an increased blast percentage may suggest transformation toward AML. Given the therapeutic and prognostic implications—where MDS and AML require cytotoxic or disease‐modifying therapies in contrast to nutritional deficiencies, which require only vitamin replacement—distinguishing between these entities is of paramount importance.

Published reports illustrate how severe deficiencies in vitamin B12 (cobalamin) and folate can present with hematologic abnormalities that mimic MDS and, on occasion, AML. For example, vitamin B12 deficiency alone has been documented to produce pancytopenia, macrocytosis, and increased blast‐like cells in the bone marrow that initially suggested MDS or AML before resolution with supplementation [10–17]. Similarly, case reports of severe combined vitamin B12 and folate deficiency have documented dramatic pancytopenia with macrocytosis and marrow findings that require careful interpretation to avoid unnecessary and potentially harmful treatment for presumed primary bone marrow disorders [3–5].

In this report, we describe a patient with severe folate deficiency and adequate vitamin B12 levels whose clinical presentation and bone marrow findings initially suggested a diagnosis of MDS/AML. The subsequent evaluation, including targeted nutritional testing and response to folate repletion, ultimately clarified the diagnosis and obviated unnecessary cytotoxic therapy. This case underscores the importance of considering severe nutritional deficiencies in the differential diagnosis of pancytopenia and marrow dysplasia, emphasizing that even dramatic clinical and morphologic features may sometimes reflect a reversible pathology rather than a primary malignancy.

## 2. Case Presentation

A 56‐year‐old female with a past medical history significant for hypothyroidism resulting from remote total thyroidectomy, gastric sleeve placement, and malnutrition was transferred from an outside hospital with severe pancytopenia concerning for aplastic anemia. She received one unit of packed RBCs at the outside institution prior to transfer. At the time of transfer to our institution, she received cefepime, metronidazole, and fluconazole for presumptive colitis and mucositis. The physical examination was unremarkable. Laboratory results revealed hemoglobin of 7.7 g/dL (normal 12.6–15.9 g/dL), hematocrit of 22.5% (normal 36.0%–49.0%), MCV of 92.2 fL (normal 81.9–101.0 fL), platelet count of < 6 k/L (normal 159–439 k/uL), and WBC of 0.33 k/uL (normal 4.30–11.30 k/uL) with severe neutropenia (absolute neutrophil count of 0.06 k/uL).

A peripheral blood smear drawn on the day of admission showed RBCs with minimal anisopoikilocytosis, increased eosinophils (50% of leukocytes) with frequent atypical nuclear segmentation, and rare hypogranular neutrophils. A concurrent peripheral blood flow cytometric evaluation demonstrated increased eosinophils (54% of viable leukocytes) and slightly increased myeloid blasts (1.5% of viable leukocytes) without abnormal B‐cell, T‐cell, NK‐cell, or mast cell populations.

**FIGURE 1:**
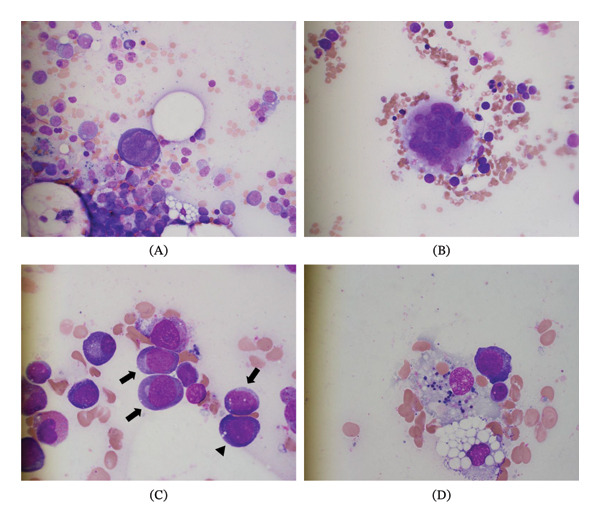
Morphologic findings in bone marrow aspirate. (A) Dyserythropoesis including megaloblastic changes and irregular nuclear features. (B) Dysmegakaryopoiesis including atypical nuclear lobation. (C) Increased blasts (denoted by arrows) with amphophilic‐to‐basophilc cytoplasm, fine chromatin, prominent nucleoli, cytoplasmic vacuoles, and rare cytoplasmic granules. Also visible is a pronormoblast (denoted by arrowhead) with darker basophilic cytoplasm and cytoplasmic vacuoles. (D) Histiocyte with hemophagocytosis.

**FIGURE 2:**
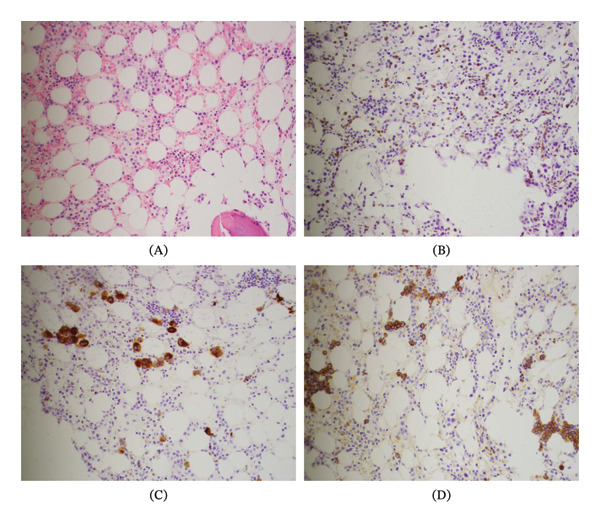
Immunohistochemical findings in bone marrow core biopsy. (A) Hematoxylin and eosin staining of the core biopsy demonstrating a marrow cellularity of approximately 20%. (B) CD34 staining demonstrates increased myeloid blasts estimated at approximately 15% of marrow cellularity. (C) CD61 staining demonstrates increased megakaryocytes including numerous small forms and forms with atypical nuclear features. (D) E‐cadherin staining demonstrates increased immature erythroid precursors including megaloblastic forms.

**FIGURE 3:**
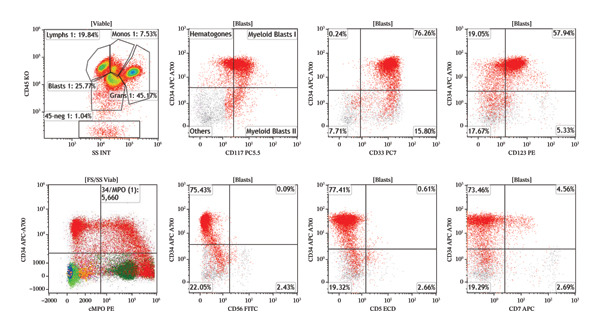
Flow cytometric immunophenotype of myeloid blasts. No definitive immunophenotypic aberrancies were detected. The blasts were positive for CD34, CD13, CD33, CD38, CD117, CD123, HLA‐DR, and MPO and were negative for CD3, CD5, CD7, CD10, CD14, CD19, CD56, CD64, and TdT.

A bone marrow biopsy performed on the day of admission demonstrated a hypocellular marrow (20% marrow cellularity) with erythroid‐predominant trilineage hematopoiesis, 12% blasts by morphology, 15% eosinophils by morphology, dyserythropoiesis, and dysmegakaryopoiesis. Erythroid maturation was left‐shifted with increased pronormoblasts and basophilic normoblasts. There were frequent erythroid precursors with megaloblastic changes, cytoplasmic vacuolization, nuclear contour irregularities, nuclear budding, macronuclei, and/or multinucleation (Figure 1A). There were frequent small, hypolobate megakaryocytes, megakaryocytes with hyperlobate nuclei, and megakaryocytes with widely spaced nuclear lobes (Figure 1B). Granulocytes demonstrated a prominent left shift with increased blasts characterized by amphophilic‐to‐basophilic cytoplasm, a high nuclear‐to‐cytoplasmic ratio, immature chromatin, variably prominent nucleoli, and sparse red cytoplasmic granules (Figure 1C). In addition, occasional histiocytes with hemophagocytosis were observed (Figure 1D). An iron stain performed on the bone marrow aspirate demonstrated increased storage iron and adequate sideroblastic iron. Ring sideroblasts were not detected. Hematoxylin and eosin (H&E) staining of the bone marrow core demonstrated a normocellular marrow (Figure 2A). Immunohistochemical staining confirmed an increased blast count (Figure 2B) and increased dysplastic megakaryocytes (Figure 2C). Increased immature erythroid cells were also noted (Figure 2D). Flow cytometric evaluation of the bone marrow aspirate specimen demonstrated increased myeloid blasts (18% of viable leukocytes) without immunophenotypic aberrancy and increased eosinophils, representing 45% of viable leukocytes (Figure 3). The morphologic findings were highly suspicious for myelodysplastic neoplasm with increased blasts 2 (MDS‐IB2) per the World Health Organization (WHO) 5th ed. classification system or myelodysplastic syndrome/AML (MDS/AML) per the International Consensus Conference (ICC) 2022 classification system. Both MDS‐IB2 and MDS/AML are associated with an aggressive disease course and higher rate of progression to frank AML. Depending upon the mutational and cytogenetic profile, the median overall survival of high‐risk MDS is approximately 18 months in the absence of curative therapy [18, 19].

Acute erythroblastic leukemia (formerly called pure erythroid leukemia) was also considered given the erythroid predominance and left shift, but this entity typically presents with > 80% erythroid precursors and > 30% pronormoblasts in the marrow, which were not seen in this case. In addition, acute erythroblastic leukemia is associated with a high incidence of biallelic *TP53* mutations, which were not seen in this case. The presence of increased eosinophils also raised the possibility of a myeloid neoplasm with eosinophilia and tyrosine kinase gene fusions, but subsequent cytogenetic studies were not supportive of this diagnosis.

One day following the bone marrow biopsy, laboratory studies demonstrated a serum folate level of 2.6 pg/mL (normal ≥ 5.9 pg/mL) with adequate vitamin B12 (> 1500 pg/mL, normal 180–914 pg/mL). Additional studies demonstrated normal serum iron (103 μg/dL, normal 28–170 μg/dL), decreased TIBC (159 μg/dL, normal 240–450 μg/dL), increased transferrin saturation (65%, normal 20%–50%), increased ferritin (1531 ng/mL, normal 13–150 ng/mL), and normal serum copper (86 μg/dL, normal 80–155 μg/dL). Subsequent ancillary studies performed on the bone marrow specimen, including cytogenomic single nucleotide polymorphism (SNP) microarray, karyotype, eosinophilia panel fluorescence in situ hybridization (FISH), AML panel FISH, FISH for BCR::ABL1, and rapid AML targeted therapy mutation panel by next generation sequencing (NGS), demonstrated no abnormalities. Myeloid malignancies mutation and copy number variation panel by NGS demonstrated one Tier 1 variant in DNMT3A with a variant allele frequency (VAF) of 1.2%, as well as a Tier 2 variant of unknown clinical significance in KMT2A with a VAF of 49.6%. Taken together, these findings were more suggestive of a reactive marrow process or marrow regeneration in the setting of severe folate deficiency, rather than an advanced myeloid neoplasm.

Folate supplementation was initiated, and the patient’s cytopenias normalized over the next 18 days. A follow‐up bone marrow biopsy performed at that time demonstrated a normocellular marrow (60% marrow cellularity) with myeloid‐predominant trilineage hematopoiesis, mild dysmegakaryopoiesis, and no increase in blasts (1% by morphology). Morphologic evaluation of the bone marrow aspirate showed increased small, hypolobate megakaryocytes and rare erythroids with nuclear contour irregularities. Both the dysmegakaryopoiesis and the erythroid atypia were improved in comparison to the prior bone marrow biopsy. Given that dysplasia and increased blasts can be seen in the setting of severe nutritional deficiency (among other non‐neoplastic processes), the lack of cytogenetic and molecular support for a myeloid neoplasm, and improvement in cytopenias with folate repletion, the overall findings were felt to be most consistent with an unusual reactive process in the setting of folate deficiency.

## 3. Discussion

This case highlights a rare but clinically significant presentation of severe folate deficiency mimicking an advanced myeloid neoplasm. While folate deficiency is a well‐recognized cause of megaloblastic anemia [6], its ability to produce profound pancytopenia with bone marrow dysplasia and increased blasts closely resembling MDS or even AML is uncommon and diagnostically challenging. The dramatic morphologic abnormalities observed in this patient—particularly the dysmegakaryopoiesis, dyserythropoiesis, left‐shifted granulopoiesis, and increased blast percentage—underscore the importance of considering reversible nutritional deficiencies in the differential diagnosis of a suspected myeloid malignancy.

Folate is essential for DNA synthesis and cell division, and its deficiency leads to impaired thymidylate and purine synthesis, resulting in ineffective hematopoiesis. These pathophysiologic mechanisms account for the classic megaloblastic changes seen in erythroid precursors, including increased cell size, nuclear irregularities, multinucleation, and nuclear–cytoplasmic asynchrony [8, 20]. In severe cases, these abnormalities extend beyond the erythroid lineage, affecting granulocytic and megakaryocytic maturation and giving rise to multilineage dysplasia and pancytopenia [8]. The bone marrow may be hypercellular or hypocellular, further complicating the distinction from MDS or aplastic anemia.

The increased blast percentage observed in this case is particularly noteworthy. Although blasts are a defining feature of both MDS with increased blasts and AML, prior reports have documented transient increases in morphologically immature precursor cells in the setting of severe folate and/or vitamin B12 deficiency [3–5, 10–17]. This likely reflects ineffective hematopoiesis and regenerative stress rather than true leukemic transformation [21]. Importantly, these “blast‐like” cells typically lack reproducible immunophenotypic aberrancies [22] and do not harbor the cytogenetic or molecular abnormalities characteristic of clonal myeloid neoplasms, although there are rare reports of reversible cytogenetic abnormalities in the setting of nutritional deficiency [23, 24]. In the present case, extensive cytogenetic and NGS studies failed to demonstrate definitive evidence of a myeloid malignancy, supporting a reactive etiology.

The detection of a low‐level DNMT3A mutation (VAF: 1.2%) further illustrates the diagnostic complexity of this case. Clonal hematopoiesis of indeterminate potential (CHIP) is increasingly recognized in otherwise healthy individuals and can coexist with non‐neoplastic conditions, including nutritional deficiencies [25]. Although DNMT3A mutations are common in MDS, the low VAF and the absence of corroborating cytogenetic or clinical evidence of disease argue against a diagnosis of MDS in this patient. This emphasizes that the molecular findings must be interpreted in the context of clinical findings and should not be used in isolation to establish a diagnosis of a myeloid malignancy.

The patient’s history of gastric sleeve surgery is a key predisposing factor, as bariatric procedures are associated with impaired absorption of folate and other micronutrients [26]. Folate deficiency can develop relatively rapidly due to limited body stores, particularly in patients with altered gastrointestinal anatomy or inadequate supplementation [27]. This case, therefore, also highlights the importance of a thorough clinical history and early nutritional assessment in patients presenting with unexplained cytopenias.

In addition to nutritional deficiencies, there are several other non‐neoplastic processes that can cause increased blasts and/or dysplasia. Increased blasts can be seen in the setting of sepsis, acute trauma, and autoimmune/inflammatory disorders, among other causes [28]. In these settings, the increase in blasts is typically mild, and the blasts lack significant immunophenotypic abnormalities by flow cytometry. Dysplasia can be seen with infection, medication effects, toxin exposure, chronic inflammation, and stress. For example, infection with human immunodeficiency virus type 1 (HIV‐1) can lead to dyspoietic features in granulocytic, erythroid, and megakaryocytic lineages that can mimic MDS [29, 30]. It is imperative that these potential causes of increased blasts or dysplasia are excluded before rendering a diagnosis of a myeloid neoplasm.

The rapid and near‐complete normalization of peripheral blood counts and bone marrow morphology following folate repletion provides strong evidence for a causal relationship between folate deficiency and the observed hematologic abnormalities. Such reversibility is inconsistent with primary MDS or AML and serves as a critical diagnostic clue. Several prior case reports and small series have documented similar reversibility of dysplastic marrow findings after correction of folate and/or vitamin B12 deficiency [3–5, 10–17], reinforcing the principle that nutritional deficiencies must be excluded before making a definitive diagnosis of MDS, particularly in cases lacking cytogenetic or molecular support.

While severe folate deficiency typically presents with macrocytic anemia, in the present case, it presented with a normocytic normochromic anemia. This was likely the result of numerous coexisting conditions, including underlying chronic disease (hypothyroidism), recent transfusion, severe malnutrition associated with protein deficiency, and *Pneumocystis jirovecii* pneumonia (PJP) that was diagnosed three days after the initial bone marrow biopsy and required intubation for acute hypoxic respiratory failure. Together, these confounding factors likely contributed to a normocytic anemia rather than the expected macrocytic anemia. Although rheumatologic testing was unrevealing, the possibility of an additional chronic disease could not be excluded.

In summary, this case underscores that severe folate deficiency can closely mimic high‐risk MDS or MDS/AML both clinically and morphologically, including the presence of pancytopenia, multilineage dysplasia, and increased blasts. Awareness of this diagnostic pitfall is essential to avoid unnecessary and potentially harmful cytotoxic therapy. Comprehensive evaluation, including nutritional studies, cytogenetic and molecular testing, and careful assessment of clinical response to vitamin repletion, is crucial in distinguishing reversible nutritional disorders from true clonal myeloid neoplasms.

## Author Contributions

Scott Potter: writing–review and editing and investigation.

Margaret Williams: writing–review and editing and investigation.

Timothy M. Hanley: supervision, writing–original draft, writing–review and editing, investigation, visualization, and conceptualization.

All authors contributed to the clinical management of the patient, data collection, and manuscript preparation.

## Funding

This study has no funding source.

## Disclosure

All authors have read and approved the final version of the manuscript. The corresponding author, Timothy M. Hanley, had full access to all of the data in this study and takes complete responsibility for the integrity of the data and the accuracy of the data analysis.

## Ethics Statement

The University of Utah Institutional Review Board does not require ethical approval of case reports that do not meet the definition of human subject research.

## Conflicts of Interest

The authors declare no conflicts of interest.

## Supporting Information

Additional supporting information can be found online in the Supporting Information section.

## Supporting information


**Supporting Information** Completed CARE checklist.

## Data Availability

The data that support the findings of this study are available from the corresponding author on reasonable request. The data are not publicly available due to privacy or ethical restrictions.
